# Serological Investigations on Environmental Allergens Triggering Allergic Dermatitis in Dogs from Western Romania

**DOI:** 10.3390/vetsci12040337

**Published:** 2025-04-05

**Authors:** Alexandra Ban-Cucerzan, Diana Obistioiu, Kalman Imre, Adriana Morar, Tiana Florea, Sebastian-Alexandru Popa, Răzvan-Tudor Pătrînjan, Miruna Șerdean, Emil Tîrziu

**Affiliations:** 1 Faculty of Veterinary Medicine, University of Life Sciences “King Mihai I” from Timisoara, 300645 Timisoara, Romania; kalmanimre@usvt.ro (K.I.); adrianamorar@usvt.ro (A.M.); tijana.florea@usvt.ro (T.F.); razvan.patrinjan.fmv@usvt.ro (R.-T.P.); miruna.serdean.fmv@usvt.ro (M.Ș.); emiltirziu@usvt.ro (E.T.); 2 Research Institute for Biosecurity and Bioengineering, University of Life Sciences “King Mihai I” from Timisoara, 300645 Timisoara, Romania; 3 Faculty of Agriculture, University of Life Sciences “King Mihai I” from Timisoara, Calea Aradului No. 119, 300645 Timisoara, Romania; dianaobistioiu@usvt.ro

**Keywords:** allergies, dogs, atopic dermatitis, serological tests

## Abstract

This study focused on identifying the environmental allergens causing allergic dermatitis in 250 dogs from Western Romania. Among the 250 dogs tested, 43% (107) exhibited significant allergic reactions (IgE levels greater than 2 kU/L), particularly in Maltese, French Bulldogs, Golden Retrievers, and West Highland White Terriers. The highest reactivity was observed to house dust mites (*Dermatophagoides farinae*, 91%), rye pollen (45%), and flea allergen *Ctef* 1 (15%). Statistical analyses revealed significant correlations between breed, sex, and living environment. Males exhibited a higher susceptibility to allergies (*p* < 0.001), whereas dogs that spent most of their time indoors were significantly more susceptible to allergic diseases than their mostly outdoors counterparts (*p* < 0.05). Additionally, dogs under two years old, especially those on a dry food diet, had an elevated risk of developing allergies (*p* < 0.01). Clinical manifestations included pruritus (60%), otitis externa (42%), and specific skin lesions (66%). The study underscores the role of environmental and dietary factors in the development of allergies in dogs. However, financial limitations related to allergy testing kits restricted the sample size, highlighting the need for further, more comprehensive research to enhance the generalizability of these findings.

## Simple Summary

Allergic skin conditions are a common encounter in dogs. This study analyzed 250 dogs from Western Romania, identifying house dust mites (*Dermatophagoides farinae*, 91%), rye pollen (45%), and flea allergens (15%) as the most common triggers. Certain breeds, including Maltese, French Bulldogs, Golden Retrievers, and West Highland White Terriers, showed a higher predisposition to allergic reactions. Statistical analysis revealed that male dogs, indoor pets, and young dogs on a dry food diet were at a higher risk of developing allergic skin conditions. The most frequent symptoms included itching (60%), ear infections (42%), and skin lesions (66%). These findings suggest a relationship between environmental and dietary factors and inflammatory dermatoses in dogs, providing population-level insights for Western Romania. While common environmental allergens are known triggers, this study does not establish which ones are the most prevalent. Additionally, although allergen testing can aid in identifying sensitivities, further research is needed to determine its impact on patient outcomes, including the effectiveness of immunotherapy. The potential link between diet and allergic conditions also requires more investigation to establish a stronger correlation.

## 1. Introduction

In the last decade, the prevalence of allergic skin disease in dogs has increased. While this trend is likely influenced by environmental factors and improved diagnostic recognition, genetic predisposition also plays a crucial role. Genome-wide association studies have identified breed-specific polymorphisms associated with atopic dermatitis, including variations in PROM1, RAB3C, and PKP2 genes, which may contribute to individual susceptibility. However, there is no conclusive evidence that recent genetic changes have directly caused the rising prevalence of allergic skin disease in dogs [1,2]. In dogs, allergic manifestations can present clinically in various forms, such as atopic dermatitis, food-induced atopic dermatitis, allergic otitis, contact dermatitis, and allergic rhinitis [3]. A wide range of allergens can elicit these clinical manifestations, with the most prevalent being mites, pollen, molds, and pollutants [4].

Although some allergic reactions appear mild at first, they significantly increase the risk of secondary infections and complications. The presence of pruritus often prompts affected animals to scratch, bite, or lick the involved skin areas, resulting in the development of local irritations, excoriations, erythema, and sometimes even deeper lesions such as ulcerations. This behavior increases the skin’s susceptibility to infections by various bacterial agents [5].

Canine skin serves several functions, including thermoregulation, defense against harmful environmental factors, and shock absorption due to its elasticity, aiding in the animal’s integration into the environment and influencing sexual behavior through the sebaceous and sweat glands. When the protection barrier is lost, a number of imbalances begin to emerge, one of which is the related allergy, which is an overreaction against exogenous antigens that are normally well tolerated by the organism [6].

Additionally, allergic diseases such as atopic dermatitis have a consistent impact on the welfare of both affected dogs and their owners, making these diseases a very challenging task for veterinarians [7,8].

Over the years, numerous efforts have been made to develop a specific diagnostic test; however, to date, the clinical presentation and the alignment of the patient with the major and minor diagnostic criteria [9] remain the gold standard for diagnosing atopic dermatitis. Similarly, for adverse food reactions, the diet trial continues to be the definitive diagnostic approach. Molecular allergy testing has made significant progress, particularly in the context of guiding therapeutic decisions, such as immunotherapy [9,10]. It is essential for the diagnosis of food hypersensitivities, which can be characterized by overlapping clinical signs, in addition to identifying key environmental allergens, including dust mites, pollen, and mold. This distinction enables veterinarians to more effectively customize treatment strategies, thereby facilitating a more personalized approach to the management of IgE-mediated conditions [5,10]. Additionally, the information provided by these tests enables pet owners to make informed lifestyle and dietary changes, thereby reducing allergen exposure and enhancing overall patient outcomes.

Considering the heavy research that is being conducted on these increasingly common skin conditions, this study aimed to identify the predominant types of allergies in the canine population of Western Romania and to determine the most common allergens associated with these conditions, via serological allergen testing methods: the Polycheck^®^ and Sensitest^®^. Another objective was to evaluate the role of intrinsic and extrinsic factors such as breed, sex, environment, and diet in the onset of allergic disorders.

## 2. Materials and Methods

This study was conducted on a total of 430 dogs, examined at the Faculty of Veterinary Medicine in Timișoara, as well as in private veterinary practices in Timișoara, Arad, Oradea, and Deva. The research spanned a period of five years, ensuring a broad dataset across multiple geographical locations.

### 2.1. Inclusion Criteria

Dogs with clinical signs of allergic dermatitis were selected for the study. Symptoms included pruritus, recurrent otitis, erythema, hyperpigmentation, and papules. Commonly affected anatomical areas included the axilla, front feet, periocular, perioral, perianal, and dorsolumbar regions. Additionally, dogs presenting with gastrointestinal symptoms associated with allergic conditions were also considered for inclusion.

Since the diagnosis of allergic dermatoses based solely on clinical signs is non-specific, differential diagnoses were systematically ruled out to improve diagnostic accuracy. Conditions such as parasitic infestations (e.g., sarcoptic mange) and bacterial and fungal infections, as well as adverse food reactions or flea bite hypersensitivity, were excluded through skin scrapings, Wood’s lamp examination and fungal cultures, cytology (direct impression smears or Scotch tape tests), and dietary trials where appropriate. The final diagnosis of atopic dermatitis was based on the fulfillment of major and minor criteria as outlined by Favrot et al. (2010) [9], which remains the gold standard for clinical diagnosis.

However, once a clinical diagnosis of canine AD is made, allergy testing can be performed to identify potential causative allergens for allergen-specific immunotherapy [11].

The study population was evaluated based on sex, age, breed, diet, living conditions, and geographical location, with a 95% confidence interval (95% CI) calculated for each reported value [12].

For the purposes of this study, dogs were categorized based on their primary living environment. Apartment dogs were considered those that spent more than 80% of their time indoors, typically in urban or suburban homes. Outdoor dogs were defined as those that spent the majority (over 80%) of their time outside, such as in gardens, yards, or rural areas, while some dogs had access to both indoor and outdoor spaces, so their classification was based on their predominant living conditions.

Dogs were also categorized based on their primary diet. Dry-fed dogs were those fed exclusively on commercial dry kibble, while mixed-fed dogs received a combination of dry, wet, and/or home-cooked foods. The study did not specifically assess raw-fed dogs. To minimize variability, dietary classification was based on feeding practices over at least three months prior to allergy testing.

Following differential diagnosis and confirmation of inclusion criteria, 250 dogs were selected for allergy testing. The aim was to detect and identify allergens responsible for allergic dermatitis using the Polycheck^®^ test (for environmental allergens) and Sensitest^®^ (Synevovet) (for food allergens. The Polycheck^®^ and Sensitest^®^ tests were selected based on their ability to detect specific allergens in canine patients. Polycheck^®^ is a monoclonal enzyme-linked immunosorbent assay (ELISA) that identifies environmental allergens by measuring allergen-specific IgE levels in serum [13]. Sensitest^®^ is a similar immunoassay designed to detect food allergens, aiding in establishing possible food-related allergens [14]. These tests complement each other by differentiating between environmental and dietary allergy triggers, providing a comprehensive approach to diagnosing allergic dermatitis in dogs. The results obtained using the Sensitest^®^ have been documented in a prior publication [15] and therefore will not be detailed in this article.

### 2.2. Collection of Samples for Allergy Testing

Five milliliters of blood was collected in order to perform the Polycheck^®^ test according to the method provided by the producer—puncturing the cephalic vein and collecting it in test tubes with a clot activator. The samples were stored at room temperature for serum expression, or centrifuged for 3–5 min at 3000 rpm. After expressing the serum, the latter was transferred to Eppendorf tubes and refrigerated at 2–8 °C. If the sampling time was extended, the serum was stored in the freezer to avoid hemolysis.

### 2.3. Polycheck^®^ Test Procedure

This test allows for the detection of the following categories of allergens: *Dermatophagoides farinae*, *Dermatophagoides pteronyssinus*, *Acarus siro*, *Tyrophaqus* spp., *Lepidoglyphus* spp., *Malassezia* spp., ragweed, birch/alder/hazelnut, plantain/willow/poplar, *Parietaria* spp., rye pollen, grass mixture, stinging nettle, lamb’s quarter, plantain, mugwort, sorrel, and *Ctenocephalides* spp. The test was conducted according to the manufacturer’s recommendations. The detection package includes 12 kits. The kit includes allergen detection solutions, a buffer to be reconstituted with distilled water and refrigerated before use, and reaction cassettes along with a frame. All solutions were brought to room temperature before use. Polycheck^®^ allergy cassettes were prepared and marked solely on their long side. The cassettes were moisturized with 1 mL of wash buffer, and the excess buffer was removed by tapping them upside down on absorbent paper. Next, 250 µL of Polycheck^®^ start solution was overlaid onto the allergy cassettes, followed by incubation for 60 s.

The cassettes were then carefully tapped upside down on absorbent paper. Subsequently, 200 µL of the respective patient’s serum was added to each cassette, and they were incubated for 60 min on a shaker, with the MTP holder placed in the middle of the shaker to maintain orientation. Afterward, the cassettes underwent three washes with 1 mL of Polycheck^®^ wash buffer, followed by incubation with 250 µL of wash buffer for 5 min on the shaker, with repetition of this step. Then, 250 µL of Polycheck^®^ anti-IgE antibody was pipetted into each cassette and incubated for 45 min on a shaker, followed by three washes with 1 mL of wash buffer. Subsequently, 250 µL of Polycheck^®^ enzyme-labelled anti-ligand was added into each cassette and incubated for 20 min on a shaker, with the washing steps repeated. Finally, 250 µL of Polycheck^®^ substrate solution was pipetted into each cassette and incubated for 20 min in the dark. After decanting and washing as previously described, the membranes were air-dried, and the Polycheck^®^ allergy cassettes were evaluated using a scanner and the Biocheck^®^ imaging software version 3.7.

The results were displayed on the computer in the form of a scintigram. The test has four reactivity classes, expressed in kU/L, as follows: class 0, negative (<0.5 kU/L, no significant IgE response detected, the dog is unlikely to be sensitized to the allergen); class 1, weakly reactive (0.5–2 kU/L, low-level IgE response, the dog may be sensitized, but the clinical relevance is uncertain and the symptoms may not necessarily be linked to the allergen); class 2, strongly reactive (2–20 kU/L, a moderate-to-high IgE response suggesting a sensitization to the allergen, with a higher likelihood of clinical symptoms); class 3–4, highly reactive (>20 kU/L, a very strong IgE response indicates that the allergen is highly likely to be a significant trigger for allergic symptoms, requiring avoidance or immunotherapy).

### 2.4. Statistical Analysis

The recorded data were statistically analyzed using the STATISTICA 8.0 program (TIBCO Software version 8.0). The employed tests were the Chi-square test, the Pearson correlation test, and the Spearman rank correlation test [16], and the graphics were obtained using Python 3.11.8 (Python Software Foundation, Wilmington, DE, USA).

## 3. Results

The study involved 430 dogs, with 48% (205/430; 95% CI 42–52) being male and 52% (225/430; 95% CI 47–57) female. Additionally, 15% of the males (65/430; 95% CI 12–18) and 20% of the females (89/250; 95% CI 17–24) were neutered or spayed. Deworming protocols, both internal and external, were inadequately performed in 41% (180/430; 95% CI 37–47) of the animals.

**Figure 1:**
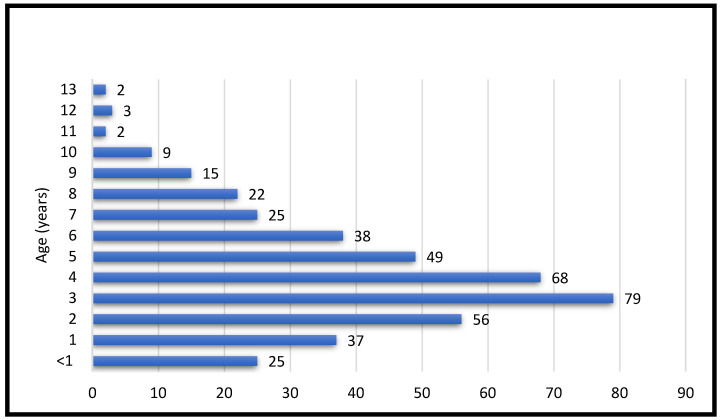
Distribution of dogs by age.

Dog breeds present in the studied population were the Akita Inu (n = 6), American Staffordshire Terrier (n = 12), Beagle (n = 15), Bichon Frise (n = 15), Bichon Havanese (n = 8), Maltese Bichon (n = 17), Boxer (n = 15), German Short-Haired Pointer (n = 4), English Bulldog (n = 9), French Bulldog (n = 30), Bullmastiff (n = 4), Bullterrier (n = 5), Poodle (n = 11), Chihuahua (n = 5), Chow-Chow (n = 2), German Shepherd (n = 46), Cocker Spaniel (n = 3), Golden Retriever (n = 16), Husky (n = 8), Labrador Retriever (n = 36), Pug (n = 11), Pekinese (n = 12), Pomeranian (n = 7), Dwarf Schnauzer (n = 6), Shar-Pei (n = 13), Shih-Tzu (n = 20), Dachshund (n = 7), Viszla (n = 9), West Highland White Terrier (n = 19), Yorkshire Terrier (n = 25), and mix breeds (n = 34). The age distribution of the canine patients ranged widely, with the youngest animals being six months old and the oldest 13 years old, with an average of 4.1 years (Figure 1).

The distribution of the 430 dogs based on their diet was as follows: 18% (78/430; 95% CI 14–22) were exclusively fed dry commercial food, while 82% (352/430; 95% CI 77–85) received a mixed diet that included dry, wet, and cooked foods.

Among the dogs receiving only dry food, ages ranged from six months to three years. The distribution was as follows: 32% (25/78; 95% CI 22–43) were below one year of age, 45% (35/78; 95% CI 33–56) were one year old, 15% (12/78; 95% CI 8–25) were two years old, and 8% (6/78; 95% CI 3–16) were three years old.

Regarding housing conditions, 64% of the dogs (275/430; 95% CI 59–68) lived in apartments, whereas 36% (155/430; 95% CI 31–40) lived predominantly outdoors.

The primary skin lesions observed in the studied population included erythema, crusts, hyperpigmentation, papules, alopecia, skin hyperplasia, lichenification, and excoriations. Red hair staining suggesting excessive licking in the foot area was present in 37% of the dogs (159/430; 95% CI 32–42).

Skin lesions were generalized in 34% of the dogs (145/430; 95% CI 29–38) and localized in 66% (285/430; 95% CI 61–70). The lesions were most frequently observed in the following areas: head (68%; 293/430; 95% CI 63–72), ears (76%; 327/430; 95% CI 71–79), neck (24%; 104/430; 95% CI 20–28), dorsal area (60%; 258/430; 95% CI 55–64), axillary region and thoracic region (50%; 215/430; 95% CI 45–54), abdomen and groin (59%; 254/430; 95% CI 54–63), and limbs (86%; 370/430; 95% CI 82–89).

Pruritus was the most common symptom among the 430 dogs. Localized pruritus affected 40% (260/430; 95% CI 56–64) and generalized pruritus affected 60% of the patients (170/430; 95% CI 34–46). Seasonal itching was observed in 3% of the dogs (15/430; 95% CI 2–6), while the remaining dogs experienced year-round irritation.

Otitis externa was detected in 42% of dogs (183/430; 95% CI 37–47), with 1% (5/430; 95% CI 1–26) presenting recurring otitis as the main and single complaint. Cytological examination of the otic discharge revealed the presence of yeast such as *Malassezia pachydermatis*, numerous rods and cocci, and a significant number of neutrophils. Otitis media, including tympanic membrane injury, was observed in 2% of the dogs (10/430; 95% CI 1–4). Additionally, conjunctivitis was found in 12% (53/430; 95% CI 9–15), vomiting in 11% (48/430; 95% CI 8–14), and diarrhea in 13% (57/430; 95% CI 10–16). Flea bite hypersensitivity affected 37% of the dogs (160/430; 95% CI 32–42) but resolved promptly following the administration of antiparasitic treatments.

### 3.1. Polycheck^®^ Test Results

Out of the 430 dogs included in the study, only 250 underwent allergy testing with the Polycheck^®^ test. A total of 107 tested positive, while the remaining 143 showed a mild test reaction.

After evaluating the database of dogs subjected to the Polycheck^®^ test, we noticed that individuals belonging to certain breeds displayed strong and high reactions, namely Maltese, French Bulldog, Golden Retriever, Labrador Retriever, Shar-Pei, and West Highland White Terrier breeds.

**Figure 2:**
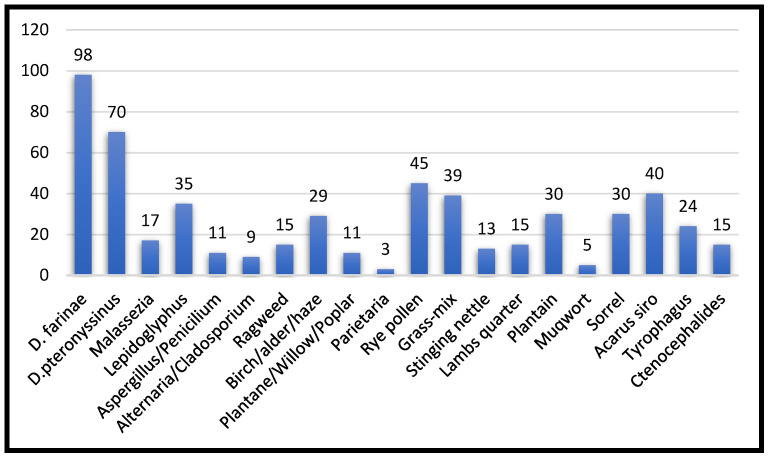
Distribution of reactivity according to allergen.

Among the 107 dogs exhibiting high reactivity to the allergens included in the Polycheck^®^ test, 18% (20/107; 95% CI 12–27) tested positive for a single allergen, with IgE levels exceeding 2 kU/L, categorized as “high reactivity.” In contrast, the remaining dogs exhibited reactions to multiple allergens. The distribution of positive reactions across various allergen categories is illustrated in Figure 2.

The highest reactivity was observed to the mite extracts. *Dermatophagoides farinae*, which, according to literature data, has the highest reactivity among mites, was responsible for 91% of positive reactions. The second one in terms of reactivity was *Dermatophagoides pteronyssinus* (65%), followed by *Acarus siro* (37%). *Lepidoglyphus destructor* and *Tyrophagus* sp. had similar reactivity levels (35% and 30%, respectively).

From the pollen category, rye pollen was the most common allergen, with 45% of dogs exhibiting a positive reaction for this allergen, and 15% had positive reactions to ragweed pollen. Additionally, the studied dog population exhibited an 11% reactivity towards fungal spore extracts and 15% reactivity towards flea extracts.

Significant variations in reactivity to the Polycheck^®^ test were noted depending on the locality of origin of the dogs. These variations appear to be primarily influenced by the environment in which the animals live, as well as by the specific vegetation characteristic of each area. The distribution of reactivity according to the animals’ place of origin is presented in Table 1. Overall, the registered incidence rate of environmental allergies was 18.47%.

### 3.2. Results of Statistical Correlations

The Chi-square test revealed a statistically significant association (*p* < 0.01) between breed and allergic status. Additionally, a highly significant correlation was found regarding reactivity based on the sex of the animal (*p* < 0.001), with males exhibiting a greater susceptibility to atopic dermatitis. A strong correlation was also observed between the age groups under 1 year and those between 2 and 3 years (*p* < 0.01), as well as between dogs under 1 year and those over 3 years of age (*p* < 0.01). Notably, dogs younger than 2 years are more likely to develop allergic conditions.

Furthermore, a significant difference (*p* < 0.05) was detected concerning the living environment, with indoor dogs showing evidence of differential risk, meaning they had a significantly higher likelihood of developing allergic diseases compared to dogs residing outdoors. The type of diet consumed by the dogs also showed a very significant impact (*p* < 0.01). Specifically, dogs fed a dry food diet demonstrated a higher likelihood of developing food allergies, with those under 2 years old on such a diet having a particularly elevated risk. Table 2 summarizes the key statistical findings.

**Figure 3:**
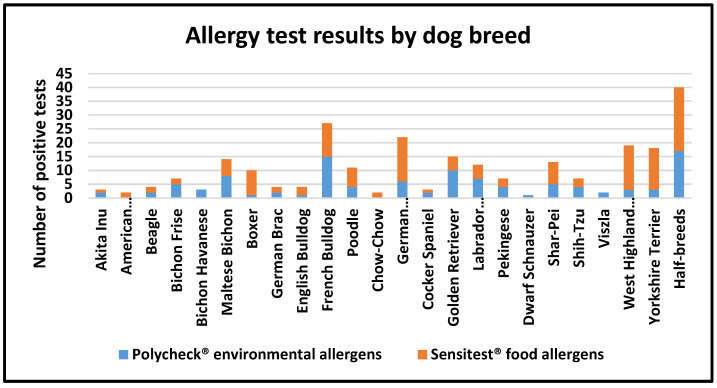
Prevalence and correlation of environmental and food allergies in different dog breeds.

A comparison between the dogs with significant sensitivity reactions to the Polycheck^®^ and Sensitest^®^ tests [15], categorized by breed, revealed that while some breeds may have a genetic tendency toward atopic allergies, in most cases, they are also prone to food-related sensitivities (Figure 3).

**Figure 4:**
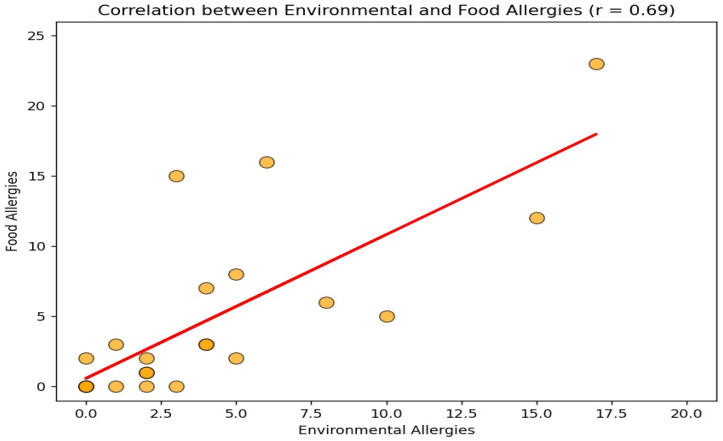
Correlation between environmental and food allergies.

The Pearson correlation coefficient between environmental allergens (Polycheck^®^) and food allergens (Sensitest^®^) was *p* < 0.05. This indicates a strong positive linear relationship between the two variables. The linear regression analysis revealed that for each additional environmental allergy, the number of food allergies was expected to increase by 0.97 on average (Figure 4).

**Figure 5:**
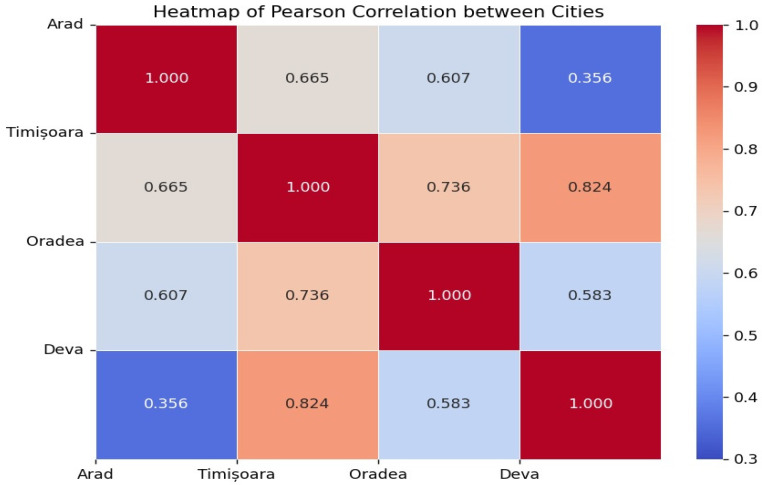
Pearson correlation between allergens in different cities.

The Pearson correlation coefficient for the allergens across the four cities (Arad, Timișoara, Oradea, and Deva) revealed a moderate positive correlation (0.665) between Arad and Timișoara, a strong positive correlation (0.736) between Timișoara and Oradea, a very strong positive correlation (0.824) between Timișoara and Deva, and a moderate positive correlation (0.583) between Oradea and Deva. Arad and Deva had the weakest correlation (0.356). This analysis shows that Timișoara and Deva have the strongest correlation in terms of allergen presence, as indicated by the similar allergen profiles. Arad and Deva showed the weakest correlation, suggesting more variability in allergen profiles between these two cities (Figure 5).

Spearman rank correlation showed a similar trend to the Pearson correlation, with Timișoara and Oradea having a strong positive correlation (0.827) and Arad and Deva having a moderate positive correlation (0.501).

## 4. Discussion

The study found a relatively high frequency of allergies among the canine population. From a total of 430 dogs, 250 were subjected to allergy testing. Among them, 107 dogs (43%) tested positive, displaying significant allergic reactions, indicating that a notable proportion of the population was affected. Research consistently indicates that dogs under the age of three are more susceptible to developing allergic diseases [9,11]. This heightened vulnerability in younger dogs is confirmed by the findings of the current study, where the strongest statistical correlations were observed in dogs between two and three years of age. These results suggest that early-life exposure to environmental or dietary allergens may play a critical role in the onset of allergic conditions during this developmental stage [17].

The findings of the current study suggest that male dogs exhibit a greater susceptibility to allergic diseases. This contrasts with most research, which indicates no significant predisposition to allergic diseases based on sex [3,18,19]. However, Harvey et al. have reported a higher predisposition in castrated male dogs to developing atopic dermatitis compared to intact females [20]. This discrepancy highlights the need for further investigation into the role of sex as a potential risk factor in canine allergic diseases.

Moreover, dogs living in indoor environments were more likely to develop allergies compared to those living outdoors, indicating that environmental exposures play a crucial role in allergy onset. This finding is consistent with previous research from Northern Italy, where atopic dogs exhibited IgE hypersensitivity reactions predominantly in response to indoor allergens (77.5%), particularly house dust mites. A smaller proportion (45.3%) reacted to outdoor allergens, but many dogs showed sensitivity to both indoor and outdoor allergens [21]. The study by Masuda et al. highlights that the environmental and seasonal factors can exacerbate atopic dermatitis in dogs, particularly in indoor environments where dust mites are prevalent [22]. Statistical tests conducted by Hakanen et al., on a group of 5722 dogs, revealed that animals living in urban areas, and in the presence of other animals, are more likely to develop allergic diseases compared to animals living in enclosed spaces and with limited access to the outside environment [23].

The study also highlights breed-specific sensitivities, identifying breeds such as the Maltese, French Bulldog, Golden Retriever, and West Highland White Terrier as exhibiting heightened reactivity to allergens, particularly house dust mites and rye pollen. These findings are consistent with previous research indicating that certain breeds possess a genetic predisposition to developing allergic diseases [24]. Specifically, German Shepherds in the United Kingdom have been demonstrated to have a genetic susceptibility to atopic dermatitis [25]. Additionally, Labrador and Golden Retrievers exhibit a 50% risk of developing atopic dermatitis, which is attributed to their genetic background [26,27].

However, while certain breeds appeared more frequently in the allergic cohort, their prevalence in the general canine population must be considered to accurately assess relative risk. According to the 2023 activity report from the Romanian Kennel Club, highly represented breeds in the study, such as Golden Retrievers, Labrador Retrievers, and French Bulldogs, are also among the most commonly registered breeds in Romania [28]. This suggests that their frequency in the allergic cohort may partly reflect breed popularity rather than an inherent genetic predisposition. Future studies should calculate relative risk by comparing breed-specific allergy rates against national breed distribution data to distinguish between true genetic susceptibility and breed popularity bias.

Genetic and phenotypic traits play a crucial role in predisposing certain dog breeds to allergic responses, ranging from mild to severe. These traits influence skin barrier integrity, immune function, coat type, and anatomical structure, ultimately affecting the severity, chronicity, and treatment resistance of allergic manifestations. More specifically, breeds like Shar Pei, French Bulldog, Pug, English Bulldog, and West Highland White Terrier are known for their allergic susceptibility due to their skin structure, which creates a warm, moist microenvironment ideal for bacterial and fungal proliferation [29,30,31,32,33,34,35].

Other breeds, like the Chinese Crested, Yorkshire Terrier, Dachshund, and Pekingese, have a coat density and structure that influences how effectively their skin is protected from environmental allergens. Their thin and fine coats facilitate a direct skin exposure to allergens, leading to intense hypersensitivity reactions [36,37,38,39,40,41].

Certain breeds, like Cocker Spaniel, Labrador Retriever, Golden Retriever, Pug, and Shar Pei, are genetically predisposed to otitis externa, which is frequently triggered by allergic dermatitis. The Cocker Spaniel and the Labrador Retriever have narrow, pendulous ear canals that retain moisture and allergens. The Shar Pei has a thickened skin and a narrow ear canal that causes recurrent infections that are often resistant to conventional treatment and require surgical intervention. In Pugs, their brachycephalic anatomy contributes to poor ventilation of the ear canal, making it suitable for yeast and bacterial infections, often triggered by allergies [29,30,32,42,43]. Table 3 summarizes some genetic traits that influence the severity of the allergies.

Collectively, these observations underscore the significant role of genetic factors in the prevalence and severity of allergic conditions across various canine breeds.

The current study also found a significant correlation between environmental factors, diet, and the development of allergies. Dogs consuming dry food, especially those under two years of age, were more prone to developing food allergies, supporting the notion that dietary components might influence allergic predispositions.

The study reveals several important findings related to the symptoms and clinical manifestations of allergic diseases in the dog population in Western Romania.

Pruritus, or itching, was confirmed as the most common symptom, affecting 60% of the studied dogs. The prevalence of both localized and generalized pruritus highlights the significant discomfort experienced by allergic dogs, with many dogs showing year-round itching rather than seasonal flares, which only affected a small percentage (3%) of the population. The skin lesions documented in the study, such as erythema, crusts, hyperpigmentation, and alopecia, are consistent with the typical signs of atopic dermatitis and other allergic skin diseases, with 34% of the dogs expressing generalized lesions, which suggests variability in the severity and extent of the allergic reactions.

Otitis externa (OE) was observed in 42% of the dogs, highlighting its frequent association with allergic skin diseases. While OE can occur as a secondary infection due to bacterial or yeast overgrowth, it is a multifactorial condition influenced by primary factors such as allergic dermatitis, predisposing factors (e.g., breed-related ear canal anatomy), and perpetuating factors that contribute to chronicity [38,39]. This complexity underscores the need for a comprehensive diagnostic and management approach.

The geographical variation in allergen reactivity may be influenced by differences in climate, humidity, vegetation, and urbanization across the studied regions [45]. For example, Timișoara and Deva, which showed the highest correlation in allergen profiles, are characterized by a mix of urban and suburban environments with moderate humidity levels. In contrast, Arad and Oradea, which exhibited weaker correlations, include more rural and agricultural areas, potentially exposing dogs to different environmental allergens such as crop-related pollens or storage mites. Future studies should further explore how these environmental factors contribute to regional allergy patterns.

The study identified reactivity to various pollens. Allergens such as birch pollen [46] and ragweed pollen [47], which are well known for causing severe allergies in humans, have also been identified as highly reactive in dogs. Studies have shown that birch pollen contains proteins that can trigger significant allergic reactions, not only in humans but also in sensitive dogs [48]. Similarly, ragweed pollen is a potent allergen that affects many dogs, leading to symptoms such as itching and inflammation [49]. The high reactivity of these pollens in both humans and dogs highlights the need for careful management of exposure, particularly during peak pollen seasons [50].

However, it is important to note that while these allergens were detected in dogs exhibiting allergic dermatitis signs, a definitive diagnosis of canine atopic dermatitis (cAD) was not confirmed in these cases. Therefore, while pollen sensitivity may contribute to allergic symptoms, a further diagnostic workup would be needed to establish cAD as the underlying condition.

Other studies from countries in Europe with temperate climates similar to Romania have reported similar findings. In Hungary, in a study on the prevalence of atopic dermatitis in dogs, positive intradermal tests (IDTs) revealed that 72.9% were allergic to the house dust mite *Dermatophagoides farinae*, 31.4% were sensitive to human dander, and 26% reacted to *Dermatophagoides pteronyssinus*, and among seasonal allergens, weeds (8%), common mugwort (*Artemisia vulgaris*—7.8%), and oak (*Quercus robur*—6.7%) were the most prevalent [51].

Countries with warmer climates like Greece, Italy, Spain tend to have more prolonged pollen seasons [52], especially for grasses and weeds, compared to the shorter pollen seasons in temperate regions. Olive and *Artemisia* pollens are more common allergens in Southern Europe due to different native plant species [53,54]. In Greece, Saridomichelakis et al. conducted intradermal testing on 91 dogs, obtaining the following results: 84% of the dogs reacted positively to mites, 42% to human epithelial cells, 36% to pollen, 23% to fungi, 18% to dust, and 2% to feathers [55].

In colder climates such as those in Norway, Sweden, and Finland, dust mites are less of a problem due to the cooler and drier indoor conditions during much of the year. However, *Dermatophagoides pteronyssinus* and storage mites may still be a concern, particularly in rural areas where grains are stored, attracting mites like *Acarus siro* [56].

Birch pollen is one of the most significant allergens in northern Europe, where birch forests are widespread [57].

Comprehending these regional patterns might facilitate the customization of allergy management techniques, including environmental adjustments and allergen-specific therapies, according to the dog’s geographical location and prevalent allergen exposures [58].

The study is subject to certain limitations, primarily due to the high costs associated with allergy testing kits. These financial constraints have not only restricted the sample size and frequency of testing but also highlight the need for further, more comprehensive tests. Addressing these limitations in future research could enhance the validity and generalizability of the findings.

While serological IgE testing (e.g., Polycheck^®^ and Sensitest^®^) provides valuable insights into allergen reactivity, these tests have inherent limitations. False positives can occur due to cross-reactivity, and elevated IgE levels do not always correlate with clinical disease severity Additionally, serology alone cannot distinguish between sensitization (presence of IgE without symptoms) and clinical allergy (symptomatic response to allergens) [13,59]. As such, positive test results should be interpreted alongside clinical history, intradermal testing, and elimination diets where applicable. Future research should integrate multiple diagnostic approaches to improve the accuracy of allergic disease diagnosis in dogs.

This study observed a population-wide correlation between environmental and food allergies, suggesting that dogs affected by one type of allergy may be predisposed to the other. However, it remains unclear whether this relationship is due to shared immunological mechanisms (e.g., a genetically heightened IgE response), environmental influences, or a reporting bias in symptomatic dogs. At the individual level, further analysis is needed to determine whether environmental allergies increase the risk of food allergies, or whether these conditions develop independently. Future studies should explore the clinical overlap between atopic and food allergies using standardized diagnostic criteria and controlled dietary challenges.

## 5. Conclusions

This study suggests an increased sensitivity among dogs in Western Romania to environmental allergens, particularly house dust mites and rye pollen. Flea allergens were also frequently identified in the studied population. Breeds with a predisposition to allergic diseases included Maltese, French Bulldog, Golden Retriever, and West Highland White Terrier. However, this apparent breed predisposition should be interpreted with caution, as these breeds are among the most commonly owned in Romania. Future studies should compare allergic prevalence against national breed distribution data to accurately assess relative risk.

The results indicate a significant association between breed, sex, and environmental factors in allergic susceptibility, with male dogs and those living indoors exhibiting a higher predisposition. Additionally, young dogs on a dry food diet were found to have an increased risk of developing allergic conditions, emphasizing the influence of early-life dietary and environmental exposures. Future research should incorporate more precise dietary tracking and standardized feeding classifications to clarify the potential link between diet and allergic dermatitis.

These findings underscore the need for more comprehensive allergen management strategies tailored to specific breeds and living environments to effectively mitigate allergic reactions in dogs.

## Figures and Tables

**Table 1:** Distribution of reactivity by locality.

Category of Allergens	Arad n = 12	Timișoara n = 45	Oradea n = 10	Deva n = 40
*D. farinae* (house dust mite)	10	40	8	40
*D. pteronyssinus* (house dust mite)	3	22	7	38
*Malassezia* spp. (yeast)	5	3	3	6
*Lepidoglyphus* (mite)	6	15	7	7
Aspergillus/Penicillium (fungi)	-	5	2	4
Alternaria/Cladosporium (fungi)	-	3	2	4
Ragweed	4	6	4	1
Birch/alder/hazel	3	5	5	16
Plane tree/willow/poplar	2	3	4	2
Pellitory	-	-	3	-
Rye pollen	6	15	10	14
Grass mix	9	15	7	8
Nettle	1	5	2	5
Wild spinach	-	3	2	10
Plantain	4	15	5	6
Wormwood	2	-	3	-
Sorrel	7	10	4	9
*Acarus siro* (storage mite)	6	13	6	15
*Tyrophagus* sp. (mite)	5	9	3	7
*Ctenocephalides* sp. (flea)	3	4	3	5

Legend: n = number of dogs in each locality.

**Table 2:** The Chi-square statistical findings.

Factor	Statistical Significance (*p*-Value)	Key Findings
Breed & allergic status	*p* < 0.01	Statistically significant association between breed and allergic status
Sex & atopic dermatitis	*p* < 0.001	Males are more susceptible to atopic dermatitis.
Age (under 1 vs. 2–3 years)	*p* < 0.01	Strong correlation between these age groups
Age (under 1 vs. > 3 years)	*p* < 0.01	Strong correlation between these age groups
Living environment	*p* < 0.05	Indoor dogs are more predisposed to allergic diseases.
Diet (dry food & food allergies)	*p* < 0.01	Dogs on dry food diets are more likely to develop food allergies.
Young dogs on dry food	*p* < 0.01	Dogs under 2 years old on dry food are at particularly high risk.

**Table 3:** Genetic traits influence in the allergy severity.

Breed	Genetic Risk Factor	Symptoms	References
Shar Pei	Skin folds, entropion	Deep pyoderma, chronic infections, risk of blindness	[29]
Chinese Crested	Hairless phenotype	High exposure to allergens, chronic dermatitis	[36,37]
French Bulldog	Brachycephalic anatomy	Chronic otitis externa, skin fold infections	[30,31]
Golden Retriever	High IgE levels	Severe atopic dermatitis, food allergies	[43]
Labrador Retriever	IgE hypersensitivity	Chronic skin infections, gastrointestinal issues	[43]
Cocker Spaniel	Pendulous ears	Frequent otitis externa	[42]
West Highland White Terrier	Epidermal dysplasia	Severe allergic reactions, secondary infections	[33,34,35]
German Shepherd	Weak skin barrier	Contact allergies, food intolerance	[24,44]
Yorkshire Terrier	Seborrheic dermatitis	Chronic pruritus, secondary bacterial infections	[38,39]
Pug	Brachycephalic ear structure	Otitis externa, chronic yeast infections	[30,32]
Pekingese	Tear staining, thick coat	Allergic conjunctivitis, seborrheic dermatitis	[41]
Dachshund	Follicular dysplasia	Exposed skin hypersensitivity	[40]

## Data Availability

The raw data supporting the conclusions of this article will be made available by the authors on request.
